# Lacerated Wounds

**Published:** 1844-02-10

**Authors:** 


### LACERATED WOUNDS.

Hæmorrhage does not generally follow these, coagulation having soon taken place in the orifices of the injured bloodvessels. A boy was drawn by a machine-rope to a ceiling. His arm was torn off into another apartment, and his body fell to the ground. He recovered! There was no bleeding. As nerves have been injured, guard against tetanus. In old people erysipelas may follow. This kind of wound is unfavourable for adhesion; attempt it, notwithstanding, by the first intention. Treat a lacerated wound as you would an incised wound. But against threatened tetanus or erysipelas use tepid anodyne lotions instead of the balsam dressing and cold application.—*Sir Charles Bell, from London Lancet.*

